# Exosomes: a significant medium for regulating drug resistance through cargo delivery

**DOI:** 10.3389/fmolb.2024.1379822

**Published:** 2024-07-29

**Authors:** Bixuan Ren, Xiaoqing Li, Zhihua Zhang, Sheng Tai, Shan Yu

**Affiliations:** ^1^ Department of Hepatic Surgery, Second Affiliated Hospital of Harbin Medical University, Harbin, China; ^2^ Department of Pathology, Second Affiliated Hospital of Harbin Medical University, Harbin, China

**Keywords:** exosomes, drug resistance, tumor, immune escape, tumor environment

## Abstract

Exosomes are small lipid nanovesicles with a diameter of 30–150 nm. They are present in all body fluids and are actively secreted by the majority of cells through the process of exocytosis. Exosomes play an essential role in intercellular communication and act as significant molecular carriers in regulating various physiological and pathological processes, such as the emergence of drug resistance in tumors. Tumor-associated exosomes transfer drug resistance to other tumor cells by releasing substances such as multidrug resistance proteins and miRNAs through exosomes. These substances change the cell phenotype, making it resistant to drugs. Tumor-associated exosomes also play a role in impacting drug resistance in other cells, like immune cells and stromal cells. Exosomes alter the behavior and function of these cells to help tumor cells evade immune surveillance and form a tumor niche. In addition, exosomes also export substances such as tumoricidal drugs and neutralizing antibody drugs to help tumor cells resist drug therapy. In this review, we summarize the mechanisms of exosomes in promoting drug resistance by delivering cargo in the context of the tumor microenvironment (TME).

## 1 Introduction

All structures that the cell releases into the external environment are known as extracellular vesicles (EVs) (Jia et al., 2022; Vergani et al., 2022; Jeppesen et al., 2023). Exosomes, the smallest subgroup of EVs, were originally thought to be the pathway by which cells excrete waste (Johnstone et al., 1991; Clancy and D’Souza-Schorey, 2023). Tumor-associated exosomes can transport their cargo to destination cells and cause associated phenotypic changes when released into the environment (Krylova and Feng, 2023). The generation of drug resistance in tumors involves many complicated processes, such as epigenetic modifications, changes in cell signal transduction pathways, drug metabolism, and excretion. Exosomes have the function of promoting drug resistance through material transfer (Antonarakis et al., 2014). For instance, exosomes carry drug-resistant genes and transfer them from resistant tumor cells to sensitive cells. Some molecules in exosomes (such as miRNA and lncRNA) protect drug-resistant tumor cells from treatment-induced cytotoxicity by inhibiting the apoptosis pathway, increasing tumor tolerance to chemotherapeutic drugs. Multiple exosomes were found to be upregulated in the plasma of patients with drug-resistant tumors, which suggests that different elements of exosomes influence tumor invasion and progression (Antonarakis et al., 2014). In this review, we focus on exosomes in tumor drug resistance research.

## 2 Exosomes mediate drug resistance transmission

Through horizontal transfer of genetic material, drug-resistant tumor cells can act as paracrine regulators and are abundant sources of exosomes. Exosomes can mediate the drug resistance of tumor cells by delivering a variety of cargoes like RNA (Antonarakis et al., 2014), DNA (Sansone et al., 2017), multidrug resistance (MDR)-associated proteins, and molecular metabolites such as prostaglandin E2 (PGE2) and TGF-β (Xiang et al., 2009). Exosomes play a role in transferring these cargoes with well-defined resistance phenotypes into sensitive cancer cells to alter cell growth and induce anti-preapoptotic pathways (Milman et al., 2019; Li et al., 2020a).

### 2.1 Transfer of the transmembrane protein

Chloride intracellular channel 1 (CLIC1) is an ion channel that is significantly related to drug resistance in gastric cancer cells (Ma et al., 2012). By isolating exosomes from the supernatant, Zhao et al. (2019) found that CLIC1 expression was higher in vincristine-resistant cell lines than in common GC cell lines. This proves that exosome-mediated transfer of CLIC1 can induce the development of vincristine resistance.

P-glycoprotein (P-gp) is a drug efflux pump reliant on energy regulated by the human *ABCB1* gene and genes such as *MDR1* (Lv et al., 2014). To make the drug effective, anti-tumor medications must reach the cytoplasm or nucleus. Tumor cells are able to encapsulate drugs as exosome cargo or secrete them out of the cell using P-gp. Blocking P-gp with calcium ion blockers can effectively prevent the efflux of anti-tumor drugs, thereby increasing the concentration of intracellular tumor drugs (Luo et al., 2013). Lv et al. (2014) found that the P-gp content of drug-resistant MCF-7/DOC was significantly higher than that of sensitive MCF-7/S, which indicated that tumor cells could induce chemotherapy-sensitive tumor cells to tolerate drugs by transferring P-gp.

### 2.2 Changing cytokines

LncARSR is an RNA activated in sunitinib-resistant renal cell carcinoma (RCC). It induces the expression of c-MET and AXL in RCC, which facilitates the resistance of Sunitinib by binding to miR-34/miR-449 competitively. Sunitinib resistance can be propagated by incorporating LncARSR into exosomes and delivering them to sensitive cells. Targeted nucleic acid therapy of sunitinib-resistant RCC against lncARSR or AXL/c-MET inhibitors restores the sunitinib response (Sun et al., 2018). The exosome-mediated circVMP1 delivery system has a similar effect. CircVMP1 targets the miR-524-5p-METTL3/SOX2 axis to promote cisplatin resistance in tumor cells, transmitting malignant features and cisplatin resistance to cisplatin-sensitive non-small cell lung cancer (NSCLC) (Xie et al., 2022). Analogously, microRNA-301b-3p from mesenchymal stem cells (MSCs) is delivered to chemotherapy-sensitive cells *via* exosomes to promote gastric cancer (GC) resistance by targeting TXNIP (Zhu et al., 2023). Wang et al. suggested that the knockout of the adipocyte-associated exosome LOC606724 or SNHG1 in adipocytes promotes the apoptotic effects of chemotherapy agents in multiple myeloma (MM) cells. MM cells facilitate LncRNA incorporation into adipocyte exosomes *via* methylation of LncRNA m6A mediated by METTL7A, leading to further resistance to MM (Wang et al., 2022a). In another experiment, exosomes from oxaliplatin-resistant colorectal cancer (CRC) cells delivered ciRS-122 to sensitive cells. Upregulation of miR-122 sponge and PKM2 promotes glycolysis and drug resistance. Moreover, miR-122 can target pyruvate kinase, thereby reducing the drug sensitivity of chemotherapy-sensitive cells (Wang et al., 2020a). Similarly, exosomes associated with oral squamous cell carcinoma (OSCC) induce drug resistance in tumor cells by upregulating the expression of mir-155 (Kirave et al., 2020). Liver cancer-related extracellular vesicles mediate drug efflux, thereby increasing chemotherapy drug resistance in hepatocellular carcinoma (HCC) by translocating Rab7402B (Li et al., 2020b). By downregulating miR-579-3p, circpar3-loaded exosomes induce resistance to cisplatin in the lateral population of nasopharyngeal cancer cells (Ai et al., 2023).

**FIGURE 1:**
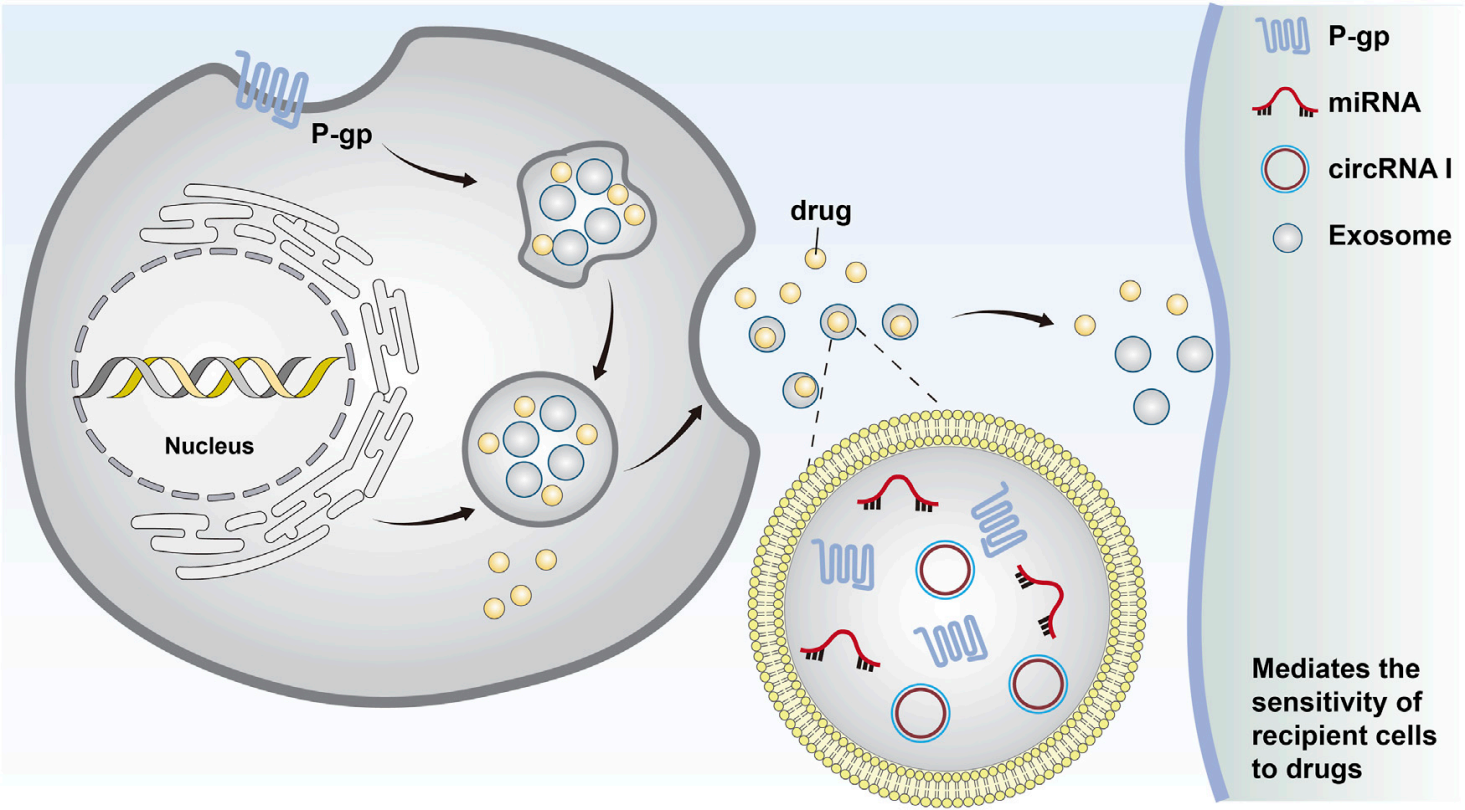
Cells form early endosomes through endocytosis. These early endosomes form the invagination of late endosomes through the plasma membrane and eventually become intracellular multi-vesticular bodies. The vesicles between 30 and 140 nm in diameter were identified as exosomes. Chemotherapeutic drugs can be excreted from cells through exosomes, and miRNAs and MDR-associated proteins can be secreted by exosomes to transfer drug resistance.

Since exosomes from cancer cells express cancer-associated cell surface proteins that sequester the substance away from the target cell, they obstruct antibody and medication therapy. For example, B-cell lymphoma cell-associated exosomes carry CD20, which binds to therapeutic anti-CD20 antibodies to deplete complement. This process protects lymphoma cells from antibody attack (Aung et al., 2011). Additionally, tumor-reactive antibodies are bound and sequestered by tumor exosomes to reduce their binding to breast cancer cells (Battke et al., 2011; Bach et al., 2017) (Figure 1).

### 2.3 Mediating ferroptosis

The generation of harmful lipid peroxides in an iron-dependent way is the cause of ferroptosis (Lei et al., 2022; Tong et al., 2022; Zhao et al., 2022). Anti-tumor drugs such as erastin block the entry of extracellular cystine into cells by inhibiting system Xc activity. This process blocks the synthesis of glutathione (GSH) in cells, weakens the oxidation resistance of cells, and eventually leads to ferroptosis (Chen et al., 2021a). Exosomes derived from NSCLC cells transfer miR-4443 to susceptible cells, conferring cisplatin resistance. Cisplatin-induced FSP1-mediated ferroptosis is inhibited by the overexpression of miR-4443 (Song et al., 2021). The researchers highlighted that the lung cancer-associated exosome circRNA101093 (cir93) regulates arachidonic acid (AA) by maintaining an increase in cir93 in lung adenocarcinoma (LUAD) cells. cir93 interacts with and increases fatty acid-binding protein 3 (FABP3). FABP3 transports AA and enhances its response to taurine, leading to a reduction in AA and the induction of chemotherapy drug tolerance (Zhang et al., 2022a). Through Zhang et al.’s investigation, these tests revealed that exosome miR-522 secreted by GC-related fibroblasts can lead to the inhibition of ALOX15. It might lessen the buildup of lipids in tumor cells to prevent ferroptosis of tumor cells, finally leading to reduced chemotherapy sensitivity (Zhang et al., 2020). Cancer-associated fibroblasts (CAFs) were found to inhibit ferroptosis and promote the chemoresistance of pancreatic ductal adenocarcinoma (PDAC) cells after gemcitabine (GEM) treatment by secreting exosomes miR-3173-5p (Qi et al., 2023). Ferroptosis provides a new direction for antagonizing tumor drug resistance. Du et al. (2021) constructed exosomes functionalized with CD47. The researchers used ultrasound to effectively load siderosis inducers into exosomes to accelerate HCC’s ferroptosis process and provide anti-tumor effects (Du et al., 2021).

## 3 Mediating immune escape

Immune escape is a significant factor in tumor drug resistance. The primary defense against cancer originates from innate immune cells, which engage in interactions with adaptive immune cells and exchange exosomes with one another (Neviani et al., 2019). Tumors exert control over pathways such as differentiation, polarization, cytokine recruitment, and others, thereby either promoting or suppressing immune-associated exosomes.

The exosomes of the cerebrospinal fluid of gliomas contain an exclusive protein called LGALS9 ligand. The ligand attaches itself to the dendritic cells’ TIM3 receptor in the cerebrospinal fluid, further interferes with the identification of dendritic cells, inhibits antigen presentation, and leads to the inactivation of cytotoxic T cell-mediated immune responses against tumors (Wang et al., 2020b). NK cells contribute to the process of immune surveillance and immune clearance of tumor cells. Lucia found that NKG2D ligands were significantly upregulated in the supernatant of exosomes derived from epithelial ovarian cancer cells. NKG2D ligands can impair the cytotoxic function of NK cells by depleting NKG2D. This suggests that exosomes containing NKG2D ligands can be released by tumors to exert cytotoxic effects (Mincheva-Nilsson and Baranov, 2014; Labani-Motlagh et al., 2016). The exosomes miR-21-5p and miR-155-5p secreted by M2 macrophages regulate the migration, growth, and invasion of glioma cells and the formation of new blood vessels (Guo et al., 2023a). Other cell populations can regulate innate immune cells through the endocrine and paracrine effects of exosomes. For instance, exosomes of adipose-derived MSCs regulate the polarization of M2 macrophages by upregulating M2 markers such as CD163, Arg1, and CD206 and activating MafB and STAT6 (Heo et al., 2019). In addition, EVs secreted by MSCs before hypoxia stimulation downregulated PTEN through miR-21-5p delivery and induced M2 polarization (Ren et al., 2019a). Wu et al. (2021a) found that exosomes produced from M2 macrophages transferred CD11b/CD18 to HCC cells. Matrix metalloproteinase-9 (MMP-9) is activated, which greatly accelerates tumor drug resistance (Wu et al., 2021a). Exosomes derived from GL261 cells significantly inhibited the amount of CD8^+^ T cells in spleen cells by blocking the breakdown of IFN-γ and granzyme B (Liu et al., 2013). Likewise, exosomes associated with NSCLC cells can inhibit CD8+T cells from secreting IFN-γ, TNF-α, and other immune cells. In addition, by binding miR-934 and upregulating the expression of protein tyrosine phosphatase 2 (SHP2), which has the Src homology 2 (SH2) domain, circUSP7 suppresses CD8+T cells (Chen et al., 2021b). Tumor cells affect the production and migration of corresponding immune cells through exosomes and reconstruct the immune microenvironment to mediate the generation of drug resistance (Table 1).

**TABLE 1:** Summary of exosome-mediated transmission of drug resistance.

Tumor type	Signal path	Resistance mechanism	Drug	Remark	Reference
Renal cell carcinoma	AXL/c-MET	Delivery of RNA	Sunitinib	MiR-34; miR-449; ncARSR	Jeppesen et al. (2023)
Non-small cell lung cancer	METTL3/SOX2	Delivery of RNA	Cis-platinum	CircVMP1; miR-524-5p	Jia et al. (2022)
Gastric cancer	TXNIP	Delivery of RNA	Cis-platinum vincristine	MicroRNA-301b-3p	Vergani et al. (2022)
Multiple myeloma	MEttL7A	Delivery of RNA	Chemotherapeutic drugs for MM	LncRNA m6A	Johnstone et al. (1991)
Colorectal cancer	PKM2	Targeting pyruvate kinase	Oxaliplatin	MiR-122	Clancy and D’Souza-Schorey (2023)
Oral squamous cell cancer	Mir-155	Promotion of EMT	Cis-platinum	Mir-155	Li et al. (2020a)
Liver cancer	—	Drug efflux	5-fluorouracil	Rab27B	Ma et al. (2012)
Nasopharyngeal cancer	SIRT1/SSRP1/MiR-579-3p	Delivery of RNA	Cis-platinum	Ebv - mir - bart4; SIRT1	Zhao et al. (2019)
Malignant lymphoma	—	Combined with therapeutic anti CD20 antibodies	Lymphoma humoral immune therapy	CD20; ATP-binding cassette (ABC) transporter A3 (ABCA3)	Lv et al. (2014)
Non-small cell lung cancer	Fsp1	Inhibition of apoptosis	Cis-platinum	MiR-4443	Luo et al. (2013)
Gastric cancer	USP7/hnRNPA1	Inhibition of apoptosis	Cis-platinum; Taxol-Phvaclitaxel TAX-PTX	ALOX15; mir −522	Sun et al. (2018)
Pancreatic cancer	ACSL4/MiR-3173-5p	Inhibition of apoptosis	Gemcitabine	MiR-3173-5p	Xie et al. (2022)
Adenocarcinoma of the lung	Cir93/FABP3	Inhibition of apoptosis	Chemotherapeutic drugs	Cir93	Zhu et al. (2023)

## 4 Tumor microenvironment

Tumor-associated exosomes mediate TME changes through endocrine effects (Attaran and Bissell, 2022), such as hypoxia (He et al., 2022), starvation, and acidosis (Xia et al., 2021; Zhang et al., 2021). These stressful environments further increase the release of tumor-associated exosomes, forming a feedback loop and ultimately promoting tumor drug resistance (Mashouri et al., 2019; Han et al., 2022).

### 4.1 Hypoxia

One study showed that miR-182-5p was markedly upregulated in the exosomes of glioblastoma in a hypoxic environment. VEGFR accumulates as a result of direct inhibition of its target Kruppel-like factors 2 and 4 by exosome miR-182-5p. It induces tumor angiogenesis. The structure of the newly formed vessels is unstable, and blood flow may be rapid, which makes it difficult for the drug to effectively cross the tumor vascular network, thereby limiting the drug’s reach to the tumor tissue and reducing its concentration within the tumor (Li et al., 2020c). At the transcriptional level, circPDK1 secreted by pancreatic cancer-associated exosome packaging can be activated by HIF1A, and the BPTF/c-myc axis is activated by miR-628-3p (Lin et al., 2022). Additionally, researchers discovered that the stronger oncogenic proteins STAT3 and FAS are present in exosomes of ovarian cancer cell lines grown in hypoxic environments, which dramatically increases *in vitro* cell motility and invasion as well as chemotherapy resistance (Dorayappan et al., 2018). Similar responses have been shown in breast cancer (BC) (King et al., 2012; Jung et al., 2018; Hannafon et al., 2019; Tian et al., 2021; Xi et al., 2021), HCC (Matsuura et al., 2019; Yu et al., 2019; Wang et al., 2022b; Yu et al., 2022; Jia et al., 2023), pancreatic cancer (Patton et al., 2020; Chen et al., 2022; Lin et al., 2022), GC (Du et al., 2020; Xia et al., 2020), CRC (Ren et al., 2019b; Li et al., 2021; Sun et al., 2021; Wu et al., 2023), and prostate cancer (PC) (Deep et al., 2020).

Critical processes in the formation of exosomes, such as vesicle budding and cargo sorting, are adversely impacted in an anoxic environment (Bister et al., 2020; He et al., 2022). Furthermore, hypoxia can affect ubiquitination proteins and ubiquitination-related enzymes, affecting the sorting of exosomal proteins. Ubiquitin proteins can accumulate through the ubiquitin-binding domains of ESCRT-0 and ESCRT-II within the multi-vesicular endosome microdomain. This process limits the number of membranes available for exosome formation, which affects exosome volume (van Niel et al., 2018; Jiang et al., 2022). In a study of PC patients, plasma from tumor patients showed higher levels of exosomes and lower volumes of exosomes. Smaller exosomes are readily able to pass through physiological barriers and enter more cells. Smaller exosomes may travel more readily through the bloodstream to the site of metastasis with this variation, which weakens the effect of drug therapy (Logozzi et al., 2021). Studies have shown that the sugar mixture on the surface of tumor-associated exosomes is highly specific (Zheng et al., 2020; Zhang et al., 2022b). The endocytosis mechanism of exosomes is sensitive to hypoxia. More exosomes can be absorbed by hypoxic cells reliant on proteoglycans (Cerezo-Magaña et al., 2021). In conclusion, modifications to plasma membrane glycoproteins brought about by hypoxia may enhance the drug resistance of tumor cells.

### 4.2 Epithelial mesenchymal transformation

Epithelial–mesenchymal transformation (EMT) of tumor tissues promotes drug-resistant cell survival. Cancer cells’ enhanced plasticity enables non-genetic adaptation to drug tolerance. Under tyrosine kinase inhibition (TKI), EMT is connected to the survival of drug-resistant cells in EGFR-mutated NSCLC (Remon et al., 2018). Exosome miR-663b secreted by cervical cancer cells directly targets the 3′-untranslated region (3′-UTR) of monoacylglycerol acyltransferase 3 (MGAT3) and participates in the process of EMT transformation. Exosome miR-663b-induced metastasis of ovarian cancer cells is inhibited by MGAT3 overexpression. At the same time, exosome miR-663b can be enucleated by cervical cancer cells, which affects the survival of drug-resistant cells (You et al., 2021). The GC-associated exosome circ133 acts on the miR-133a and RhoA axes. Circ133 promotes cancer by disinhibiting its target SIX1. Interestingly, the target SIX1 reduced the sensitivity to chemotherapy in BC cells by increasing the TGFβ signaling pathway (Micalizzi et al., 2009). Moreover, GC-associated exosomes induce the transcription of ZEB1 to regulate the differentiation of CRC cells and induce drug resistance (Ono et al., 2012). CRC-associated exosomes promote EMT and increase the intracellular accumulation of oxaliplatin by delivering miR-128-3p. They have the potential to improve drug sensitivity and reduce drug resistance, serving as novel biomarkers (Liu et al., 2022a).

### 4.3 Proangiogenic effect

**FIGURE 2:**
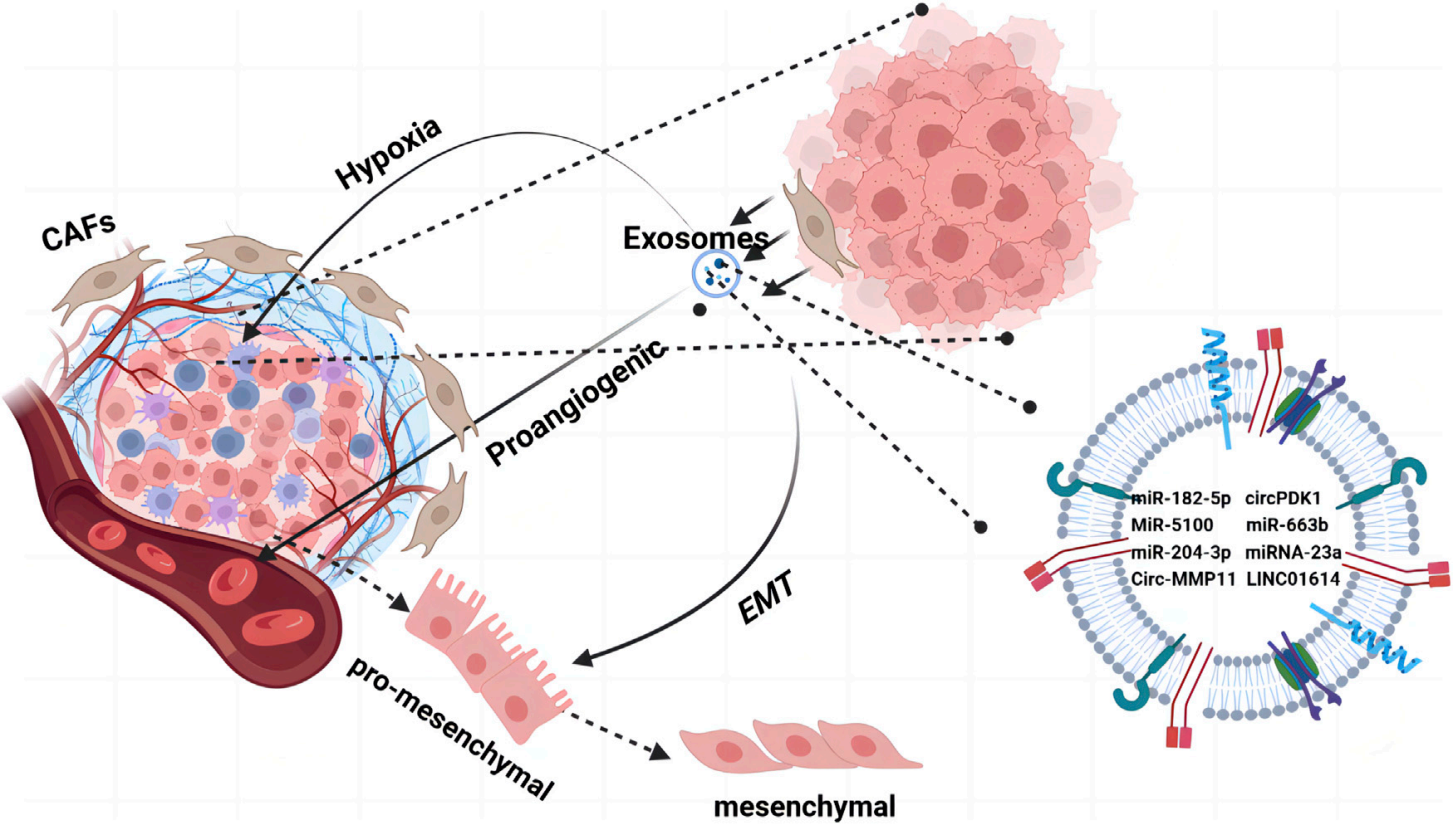
The TME orchestrates cell–cell interactions, such as paracrine and near-paracrine interactions, through tumor-associated exosome networks. It is composed of a variety of cells embedded in the extracellular matrix, including endothelial cells and tumor-associated fibroblasts. Tumor cells secrete cytokines through exosomes to promote changes in the TME such as hypoxia, angiogenesis, and EMT. Tumor cells use the regulatory mechanisms of microsystems to form a drug-resistant state.

Exosomes promote tumor angiogenesis through various mechanisms that have antagonistic effects on anti-angiogenic drugs and eventually lead to drug resistance. Exosome miR-204-3p induces vascular endothelial cells to form tubular forms *via* the ATXN1/STAT3 pathway. TAK-981 has a function in inhibiting SUMOylation. It prevents tumor growth and angiogenesis by blocking miR-204-3p′s exosome sorting process. Glioma cells can accelerate angiogenesis by upregulating SUMOylation and eliminating the inhibitory factor miR-204-3p under hypoxia (Guo et al., 2023b). OSCC cells have upregulated miRNA-210-3p to promote tumor growth by increasing tumor grade and microvascular density. Ephrin A3 expression is mechanically downregulated by the exosomal miRNA-210-3p to activate the PI3K/Akt axis. The process increases angiogenesis and advances OSCC (Wang et al., 2020c). Analogously, the high migratory potential of nasopharyngeal carcinoma cells is associated with angiogenesis. Exosome miRNA-23a inhibits the production of TSGA10 by binding to its 3′-UTR. MiRNA-23a leads to angiogenesis and increases metastasis in nasopharyngeal carcinoma (Bao et al., 2018). Exosome lncRNA may also play an essential role in regulating angiogenesis. For example, exosomal miRNA-29-3p downregulates the expression of PTEN, and the inactivated PTEN signaling pathway promotes the phosphorylation of PI3K/Akt, which promotes angiogenesis in lung cancer (Cheng et al., 2019). Angiogenin-2 (ANGPT2) is believed to promote angiogenesis by disrupting vascular stability. HCC cells can secrete exosomes containing ANGPT2. These exosomes are introduced into umbilical vein endothelial cells by intracellular action, and elevated expression of ANGPT2 causes angiogenesis and encourages drug resistance (Xie et al., 2020) (Figure 2).

### 4.4 CAF-related exosomes

Wu et al. measured RNA levels in lapatinib-resistant BC specimens and then highlighted that exosomes may be a means of delivering Circ-MMP11. The knockdown of circ-MMP11 promotes lapatinib sensitivity by inhibiting cell survival and invasion while promoting apoptosis in lapatinib-resistant BC cells (Wu et al., 2021b). In Liu’s study, cancer-associated fibroblasts of LUAD secreted a long non-coding RNA, LINC01614, by exosome packaging. This RNA directly interacts with ANXA2 and p65 to promote NF-κB activation, with the glutamine transporters SLC38A2 and SLC7A5 being overexpressed, eventually enhancing glutamine influx in cancer cells. Alterations in glutamine metabolism increase the antioxidant capacity of cells and reduce their susceptibility to drug-induced apoptosis (Liu et al., 2022b).

In previous studies, researchers have suggested that cancer stem cells (CSCs) are inherently resistant to chemotherapy-induced cell death. After chemotherapy treatment, fibroblast-derived conditioned medium (CM) promoted the proportion of CSCs (CD133+ and TOP-GFP+) and tumor viability. Further studies have shown that exosomes isolated from CM have similar effects described above (Ren et al., 2018; Hu et al., 2019). Correspondingly, through the METTL3/miR-181d-5p axis, METTL3 decreases the susceptibility of CRC cells to 5-fururacil (5-FU) and enhances exosome miR-181b-5p in CAFs (Pan et al., 2022).

## 5 Therapeutic strategies

Because of their role in promoting drug resistance in tumor cells, exosomes have great potential to be a therapeutic target for inhibiting tumor drug resistance. Limiting the biogenesis and release of exosomes or downregulating the uptake of exosomes by target cells has functions in restoring the sensitivity of tumors to chemotherapeutic drugs. For ESCFRT-dependent cells, downregulation of hepatocyte growth factor-regulated tyrosine kinase substrate (HRS) and signal transducer adapter molecule 1 (STAM1) can limit the secretion of exosomes and limit the efflux of drugs from tumor cells (Colombo et al., 2013). In addition, antagonizing drug efflux and changing the TME can indirectly compensate for the drug resistance caused by exosomes to a certain extent. For example, Diannexin can impair the angiogenesis of A431 squamous carcinoma in SCID mice by downregulating exosomes (Al-Nedawi et al., 2009). Exosomes can also induce drug killing in other ways. Autophagy and exosomes can compensate for each other in different environments to promote the export of signaling molecules (Deng et al., 2019). The combined use of chemotherapeutic drugs and autophagy inhibitors such as chloroquine or hydroxychloroquine can significantly enhance the sensitivity of chemotherapeutic drugs (Singh et al., 2018). RNAs from exosomes could compete with PD-L1 to modulate DNA damage responses, thereby enhancing chemotherapy sensitivity (Tu et al., 2019). Key concepts of therapy methods using exosomes to mediate drug effects are summarized in Table 2.

**TABLE 2:** Key concepts of therapy methods using exosomes mediating drug effects.

Treatment strategy	Mechanism	Target
Reducing the drug resistance of tumors	Blocking P-gp protein delivery and reducing the efflux of anti-tumor drugs	Calcium antagonist
Improving the sensitivity of tumors to chemotherapeutic drugs	Reversing the adverse effects of tumor metabolism and restoring drug sensitivity to chemotherapy	ESCRT-0/1 HRS STAM1 TSG101
Promoting the exogenous tumor cell killing	A combination of inhibitors targeting metabolic enzymes and impairing the immune escape of tumor cells	NKG2D
Other mechanisms that promote synergistic drug killing	Promoting tumor autophagy	ATG16L1/ATG5
	DNA damage repair	PD-L1
Antagonizing the changes in the TME, such as hypoxia, angiogenesis, and EMT	Restoring the original state of the TME and reducing the resistance to drugs	Mir-128-3p Mir-204-3P

## 6 Conclusion

The diversity of exosomes confers significant advantages as biomarkers, and their stable structure effectively transfers the genetic information of the parent cells. Tumor-derived exosomes also profoundly affect the process of drug resistance. In tumor patients, exosomes generated from tumors transfer RNA and proteins to mediate drug resistance. At the same time, tumor cells influence the TME through exosomes, causing a series of changes, such as hypoxia, to induce drug resistance. This means that the use of exosomes can reverse the drug resistance of tumor cells and increase the effectiveness of drug therapy. Since exosomes are multi-purpose carriers of communication between multiple organs, restoring their normal secretion plays a particularly important role in the treatment of patients with tumors (Dixson et al., 2023). Specific components in exosomes, such as miRNAs or proteins, may become biomarkers of tumor resistance. By examining the expression levels of specific components in extracellular vesicles secreted from patient samples, we can help predict patient response to drug therapy and guide an individualized treatment plans (Fitts et al., 2019; Hoshino et al., 2020). Targeting specific signaling pathways or related molecules involved in the generation and release of extracellular vesicles could inhibit tumor drug resistance (Pu and Ji, 2022). Exosomes are expected to be useful indicators for tumor therapy as they regulate many aspects of heterotypic cell-to-cell interactions in the TME. Plenty of exosomes have been discovered and uploaded to the Vesiclepedia online compendium. Researchers can review the identified RNA molecules and proteins in exosomes (Kalra et al., 2012). A deeper understanding of the biogenesis of tumor-associated exosomes is of great significance for further exploration of the information exchange between tumor and normal cells and for utilizing their properties to guide combined clinical therapy (Debbi et al., 2022; Rezaie et al., 2022).
